# Prognostic Value of Circulating IGFBP2 and Related Autoantibodies in Children with Metastatic Rhabdomyosarcomas

**DOI:** 10.3390/diagnostics10020115

**Published:** 2020-02-20

**Authors:** Elena Poli, Angelica Zin, Manuela Cattelan, Lucia Tombolan, Ilaria Zanetti, Angela Scagnellato, Paolo Bonvini, Gianni Bisogno

**Affiliations:** 1 Department of Woman’s and Children’s Health, Hematology and Oncology Unit; University of Padua, via Giustiniani, 3, 35128 Padua, Italy; lucia.tombolan@unipd.it (L.T.); ilaria.zanetti@unipd.it (I.Z.); angela.scagnellato@unipd.it (A.S.); gianni.bisogno@unipd.it (G.B.); 2 Institute of Pediatric Research (IRP), Fondazione Città della Speranza, Corso Stati Uniti, 4F, 35127 Padua, Italy; zin.angelica.gin@gmail.com (A.Z.); paolo.bonvini@unipd.it (P.B.); 3 Department of Statistical Sciences, University of Padua, via Cesare Battisti, 241, 35121 Padua, Italy; manuela.cattelan@stat.unipd.it

**Keywords:** rhabdomyosarcoma, pediatric soft tissue sarcomas, plasmatic biomarkers, diagnostic and prognostic factors, tumor-associated antigens, tumor-associated autoantibodies, metastatic tumors, IGFBP2

## Abstract

Insulin-like growth factor-binding protein 2 (IGFBP2) is a tumor-associated protein measurable in patients’ biopsies and blood samples. Increased IGFBP2 expression correlates with tumor severity in rhabdomyosarcoma (RMS). Thus, we examined the plasmatic IGFBP2 levels in 114 RMS patients and 15 healthy controls by ELISA assay in order to evaluate its value as a plasma biomarker for RMS. Additionally, we looked for the presence of a humoral response against IGBFP2 protein measurable by the production of anti-IGFBP2 autoantibodies. We demonstrated that both circulating IGFBP2 protein and autoantibodies were significantly higher in RMS patients with respect to controls and their combination showed a better discriminative capacity. IGFBP2 protein identified metastatic patients with worse event-free survival, whereas both IGFBP2 and anti-IGFBP2 antibodies negatively correlated with overall survival. Our study suggests that IGFBP2 and anti-IGFBP2 antibodies are useful for diagnostic and prognostic purposes, mainly as independent negative prognostic markers in metastatic patients. This is the first study that reports a specific humoral response in RMS plasma samples and proves the value of blood-based biomarkers in improving risk assessment and outcome of metastatic RMS patients.

## 1. Introduction

Rhabdomyosarcoma (RMS) is an aggressive pediatric tumor arising from soft tissues and sharing aspects with skeletal muscle lineage.

Although a rare disease, RMS comprises about three percent of childhood cancers and is the most common soft tissue sarcoma in children, with an overall incidence rate of approximately 4.5 patients per million individuals aged less than 20 years [1]. RMS is composed by a heterogeneous group of histological subtypes with different clinical and genetic features. According to the World Health Organization (WHO) RMS can be divided into four subtypes: alveolar (ARMS, about 20%), embryonal (ERMS, 60–70% of all RMS), pleomorphic, and spindle cell/sclerosing RMS (PRMS and SRMS, respectively, which comprise the remaining 10%) [2]. ERMS occurs in children under 10 years age, with a bimodal age distribution with the largest peak between 0–5 years. It typically arises in favorable sites such as head and neck, the genitourinary tract and the retroperitoneum. ARMS affects predominantly adolescents and young adults and it usually localizes to the extremities and the trunk [3]. ERMS has a relatively favorable prognosis, with 5-year survival rate of 70–80%, while ARMS is associated with a poorer prognosis, with a 5-year failure–free survival of 65%, because of its propensity for early and wide dissemination and poor response to chemotherapy.

ARMS tumors typically harbor recurrent chromosomal translocations. The most common of these is t (2;13) (q35;q14) resulting in the expression of the oncogenic fusion protein PAX3-FOXO1 composed of the paired box protein 3 (PAX3) 5’-end DNA-binding domain fused with the 3’-end transcriptional activation domain of forkhead box protein O1 (FOXO1). The PAX3-FOXO1 fusion protein can be detected in about 55% of ARMS cases, while the similar translocation t (1;13) (p36;q14), that fuses the PAX7 5’-end DNA-binding domain to FOXO1, occurs in a further 22% of ARMS patients [4,5]. ERMS tumors at the molecular level are not characterized by specific chromosomal translocations, but by a more severe genomic instability, complex karyotypes, and recurrent somatic mutations [6]. 

Multimodal therapy has improved significantly the survival of RMS patients, which now exceeds 70% for children with localized disease. Unfortunately, metastases are present in approximately 20% of RMS cases at diagnosis, causing a dramatic fall of the overall cure rate to less than 30%. Outcome, however, is not uniformly poor in metastatic patients, as it may vary significantly (5–50%) based on the type and number of the adverse factors involved [7]. To improve risk assessment and better tailor the treatment is paramount to identify biomarkers able to predict and monitor growth, dissemination, and response to therapy. Plasmatic tumor-associated antigens (TAAs) and related tumor-associated autoantibodies (TAABs) are both considered potential cancer biomarkers. Tumor antigens reflect changes in the expression of proteins likely involved in the evolutionary dynamic of cancer, whereas cancer autoantibodies sense these changes and amplify the signal when antigen expression is low [8,9,10,11,12,13,14]. 

Insulin-like growth factor binding protein 2 (IGFBP2) is one of six high-affinity insulin-like growth factor-binding proteins (IGFBP1-6) that, together with IGF receptors and the soluble factors IGF-I and IGF-II, composes the IGF system [15]. IGFBP2 is secreted into the bloodstream where it binds and modulates availability of IGF-I and -II to the target receptors [16,17]. Because of its overexpression, soluble IGFBP2 represents a good diagnostic and prognostic biomarker in several advanced cancers such as breast, glioma, ovarian, and lung cancer, eliciting a B cell-mediated immune response with the production of anti-IGFBP2 antibodies [18,19,20]. Previously, we have demonstrated that IGFBP2 is overexpressed in RMS tumors and associated with disease severity [21,22]. Herein, we evaluated whether secreted IGFBP2 represents a RMS antigen able to induce a humoral immune response leading to autoantibodies production with diagnostic and/or prognostic significance. By correlating clinical parameters and outcome data, we provided evidences that secreted IGFBP2 protein and autoantibodies can be used as blood-based biomarkers in RMS patients.

## 2. Materials and Methods 

### 2.1. Patients and Blood Specimens

A total of 114 blood samples from RMS patients enrolled in national and international pediatric sarcoma protocols RMS 4.99, EpSSG RMS 2005, and EpSSG MTS-2008 were included in this study after obtaining institutional review board approval. Biological specimens were collected from the Italian Centers participating in the protocols between July 2004 and July 2013. Studies on human samples were approved by Padua Hospital Ethics Committee (No. 191P, 20 June 2000; No. 988P, 31 March 2005). Diagnosis of all cases included in this study was reviewed by the Italian Association of Pediatric Hematology and Oncology (AIEOP) reference pathologists. The presence of tumor-specific molecular biomarkers (PAX3/7-FOXO1A and MyoD1 transcripts) was investigated by RT-PCR. The ethnicity of the patient cohort was Caucasian, equally distributed between male (*n* = 58) and female (*n* = 56) with a median age of 6.40 ± 5.97 years. All the RMS histological variants were represented: 50 alveolar RMS cases (*n* = 33, *PAX3-FOXO1+*; *n* = 7, *PAX7-FOXO1+*; *n* = 10, *PAX3/7-FOXO1-*), 55 embryonal RMS, 6 spindle cell/sclerosing and 3 pleomorphic RMS. With respect to the site of onset, 27 was arisen in a favorable site (*n* = 6, orbit; *n* = 13, urogenital non-bladder/prostate; *n* = 8, head and neck non-parameningeal), 81 in an unfavorable one (*n* = 33, head and neck parameningeal; *n* = 7, urogenital bladder/prostate; *n* = 25, extremities; *n* = 16, all “other site”), while for 6 histology was unknown. The post-surgical stage, defined according to the *Intergroup Rhabdomyosarcoma Study* IRS *Group* system, identified 3 patients belonging to IRS group I (completely-excised tumors), 9 to IRS group II (grossly-resected tumors with microscopic residual disease and/or regional lymph node involvement), 63 to IRS group III (gross residual disease after incomplete resection or biopsy), 35 to IRS group IV (metastatic disease), and for 4 of them were not known their IRS group.

In addition, 15 pediatric healthy subjects (HS; 12 males and 4 females; median age = 9.48 ± 3.75 years), hospitalized for non-oncological reasons, were included and used as controls. Peripheral blood was drawn at the time of diagnosis, prior to any type of treatment, in sodium citrate tubes and processed within 24 h. Specimens were centrifuged at 820× *g* for 10 min to separate plasma from whole blood. To avoid any type of contamination by blood cells, plasma samples were carefully harvested and transferred into fresh tubes for a further centrifugation step at 16,000× *g* for 10 min. Each plasma sample was stored as aliquots at −80 °C until use.

### 2.2. Quantitative RT-PCR

Total RNA, from 50 matched tumor biopsies selected stochastically among the 114 RMS patients enrolled in this study, was isolated using the TRIzol reagent (Invitrogen, CA, USA) and reverse transcribed using Super-Script II (Invitrogen, California, USA), according to the manufacturer’s instructions. As non-pathological normal controls fetal and adult skeletal muscle and mesenchymal stem cells were used. Quantitative RT-PCR (qRT-PCR) was performed on Viia7 thermal cycler (Applied Biosystems, CA, USA), using SYBR Green chemistry (Applied Biosystems, CA, USA) and a standard protocol of amplification. Gene-specific primer sequences were 5′-ACTCCCTGCCAACAGGAAC-3′ (forward) and 5′-GTTGGGGTTCACACACCAG-3′ (reverse) for IGFBP2, as 5′-TCCTCTGACTTCAACAGCGA-3′ (forward) and 5′-GGGTCTTACTCCTTGGAGGC-3′ (reverse) for glyceraldehyde-3-phosphate dehydrogenase (GAPDH). GAPDH was used as the normalizing reference gene. The relative expression of IGFBP2 was calculated by using the ΔΔCT method.

### 2.3. Direct ELISA Assay for Plasmatic IGFBP2 Assessment

Plasmatic levels of circulating IGFBP2 protein were determined by using the IGFBP2 enzyme-linked immunosorbent assay (ELISA) kit (RayBiotech, Inc., GA, USA), according to the manufacturer’s instructions. Each assay was performed in triplicate and the mean concentration of IGFBP2 was used for further statistical analysis.

### 2.4. Indirect ELISA Assay for Plasmatic Anti-IGFBP2 Autoantibodies Detection

Plasmatic autoantibodies (Abs) against IGFBP2 were assessed by a home-made indirect ELISA assay. Briefly, immulon 4HBX microtiter plates with extra-high binding surface (Dynex Technologies Inc., VA, USA) were coated overnight at 4 °C with human recombinant IGFBP2 protein (Sigma-Aldrich, St Louis, MO, USA) diluted in 50 mM carbonate-bicarbonate buffer (Sigma-Aldrich, St Louis, MO, USA) to a final concentration of 0.5 µg/mL. The last three columns of wells were incubated with serially diluted purified human IgG (dilution: 1:2; range: 5 ng/mL–640 ng/mL) (Sigma-Aldrich, St Louis, MO, USA) to provide standard curve. Negative control wells were not coated with IGFBP2 or IgG proteins, but only filled with carbonate-bicarbonate buffer. Plates were blocked with 1% bovine serum albumin (BSA) in 1X phosphate-buffered saline (PBS) for one hour at room temperature with gentle shaking, washed four times with 0.01% Tween-20 in PBS, and then incubated in triplicate with diluted plasma samples (1:60) in the same solution used for the blocking step, for 2 h, at room temperature with gentle shaking. After four more washes, plates were added with rabbit anti-human IgA, IgG, IgM, Kappa, Lambda-HRP (DakoCytomation, DK) diluted 1:8000 in 1% BSA in PBS 1X, for 1 h with gentle shaking. Plates were then washed and developed with 75 µL of 3,3’,5,5’-tetramethylbenzidine (TMB) (Kinkegaard and Perry Laboratories, MD, USA). The TMB substrate produced a blue color upon reaction with horseradish peroxidase conjugates that was read at a wavelength of 640 nm. Reaction was carefully monitored and stopped when the 160 ng/mL standard point reached an O.D. value of 0.3, by adding an equal volume of 1N hydrochloric acid. Plates were then read at a wavelength of 450 nm and the O.D. value of each plasma sample was obtained subtracting the O.D. of the carbonate-bicarbonate buffer-only coated wells. The final concentration of plasmatic IGFBP2 Abs was worked out from the standard curve present in each plate and multiplied by the plasma dilution factor. Each plasma sample was assessed in triplicate in three independent assays.

### 2.5. Total IgG Measurement

Concentration of total plasma IgG levels in each plasma sample was used to normalize IGFBP2 specific antibody response. Immunoglobulins were quantified by Human IgG ELISA kit (RayBiotech, Inc., GA, USA) according to the manufacturer’s instructions. All plasma samples were assessed in triplicate.

### 2.6. Statistical Analysis

All statistical comparisons were performed using non-parametric Mann-Whitney or Fisher’s exact test. A receiver-operator characteristic (ROC) curve was calculated to determine the discriminatory capacity and the cutoff values of IGFBP2 protein and Abs to predict diagnosis, event occurrence, or death with the highest sensitivity and specificity. To study the impact of the combination of IGFBP2 protein and Abs for diagnosis, ROC curves analysis was based on binary logistic regression. The area under the ROC curves was analyzed by the Hanley and McNeil method. The main outcomes considered in this study were event-free survival (EFS) and overall survival (OS). EFS was calculated from the date of diagnosis to the date of the first occurrence of disease progression, relapse after response, or death from any cause or to the date of the last follow-up. OS was calculated from the date of diagnosis until death from any cause or the last follow-up. Patients who did not experience any event were censored at their last follow-up time. Survival curves were estimated by the Kaplan–Meier method and overall differences were compared by the log-rank test. Cox uni-and multivariate proportional hazard analysis were carried out to estimate the prognostic impact of IGFBP2 protein and autoantibodies, alone or in association with other known prognostic factors both on EFS and OS. Statistical analyses were carried out using GraphPad Prism 5, SPSS 23 and R statistical software [23]. All P values were two-tailed and considered statistically significant at the alpha level of 0.05.

## 3. Results

### 3.1. Patients Characteristics

The main clinical characteristics and molecular features of the 114 patients included in the study are reported in Table 1. Median age at diagnosis was 6.40 ± 5.97 years. Survival data were available for 106 patients, with a median follow-up time of 4.08 yrs (range: 0.24–12.44 yrs). Thirty-six patients experienced relapse (local, lymph node, or distal metastasis) and 13 suffered progression of disease. Median time for event occurrence was 2.09 yrs (range: 0.1–7.74 yrs). Forty-three patients died for disease progression and one patient for treatment-related toxicity. The 5-years EFS and OS of the whole cohort of patients was 46% (95% CI = 37%–57.2%) and 58.8 % (95% CI = 49.6%–69.7%), respectively. 

### 3.2. Plasmatic IGFBP2 Protein and Anti-IGFBP2 Autoantibodies as Diagnostic Biomarkers in RMS Patients

**Figure 1:**
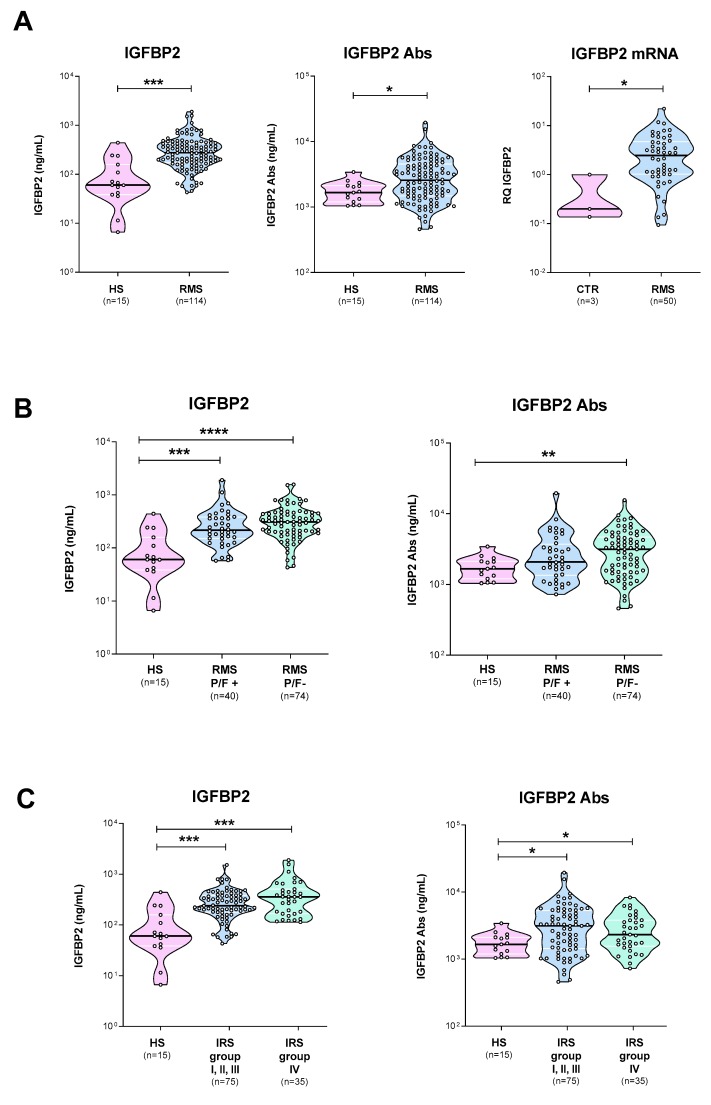
Plasmatic IGFBP2 protein and autoantibodies levels in RMS patients. (**A**) IGFBP2 protein and autoantibodies (Abs) in healthy subjects (HS) and RMS patients (RMS) (left and central panels). IGFBP2 mRNA levels in RMS patients and controls (right panel). Plasmatic levels of IGFBP2 protein and autoantibodies in RMS patients distinguished according to (**B**) fusion status or (**C**) IRS group classification. *P/F+*, *PAX3/7-FOXO1*-positive; *P/F-*, *PAX3/7-FOXO1-*negative; *FOXO1*, forkhead box protein 1; *PAX3*, paired box 3; *PAX7*, paired box 7; *p* < 0.05 (*); *p* < 0.01 (**); *p* < 0.001 (***); *p* < 0.0001 (****).

Plasmatic levels of circulating IGFBP2 protein and anti-IGFBP2 antibodies were measured at diagnosis by commercially available and home-made ELISA assays, respectively. We found that plasmatic levels of both IGFBP2 protein and autoantibodies were significantly higher in children with RMS compared to controls (IGFBP2 median: 275.5 *vs*. 60.1 ng/mL, *p* < 0.001; IGFBP2 Abs median: 2559 *vs*. 1663 ng/mL, *p* = 0.023, respectively), as was IGFBP2 mRNA (*p* = 0,024) (Figure 1A). However, while IGFBP2 protein levels were statistically significant both in *PAX3/7-FOXO1* fusion-positive (*p* = 0.0001) and –negative (*p* < 0.0001) plasma samples, anti-IGFBP2 antibodies were only in fusion-negative ones (*p* = 0.009) (Figure 1B). In addition, plasmatic levels of IGFBP2 and anti-IGFBP2 antibodies were not statistically different comparing patients with localized (IRS I, II, III) *vs*. metastatic (IRS IV) disease at diagnosis (Figure 1C). Similarly, no other known clinical parameters, including gender, age, tumor size and site were found associated with IGFBP2 and anti-IGFBP2 antibodies levels (Figure S1). 

**Figure 2:**
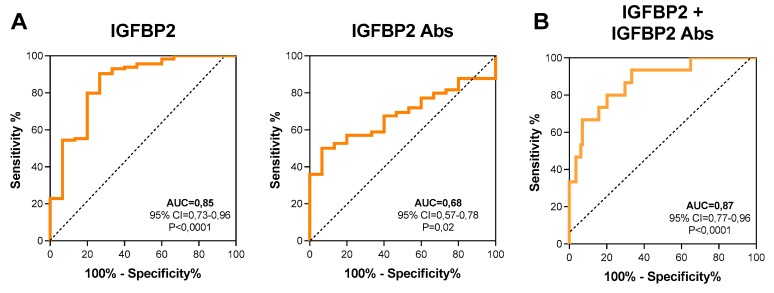
Diagnostic value of IGFBP2 protein and autoantibodies. (**A**) Receiver operating characteristic (ROC) curves of plasmatic IGFBP2 protein and autoantibodies alone or (**B**) in combination, to distinguish RMS patients from healthy subjects. AUC: area under the curve; CI: confidence interval.

In order to further discriminate between cancer patients and controls, ROC curves were generated using plasmatic values of IGFBP2 and anti-IGFBP2 antibodies. The AUC were 0.85 (95% CI = 0.736–0.969; *p* < 0.0001) and 0.68 (95% CI = 0.578−0.783; *p* = 0.02), respectively (Figure 2), with 111.3 ng/mL (sens = 90.35%; spec = 73.33%) and 2577 ng/mL (sens = 93.33%; spec = 50.00%) the corresponding cutoff values maximizing both sensitivity and specificity. ROC curve analysis performed by combining IGFBP2 and anti-IGFBP2 values increased further the discriminative power of the test (Figure 2B; AUC = 0.87; 95% CI = 0.778−0.966; *p* < 0.0001) and allowed samples to be grouped in four new clusters: low levels of IGFBP2 and high levels of anti-IGFBP2 antibodies (cluster I: 1 HS, 4 RMS), high levels of both (cluster II: 0 HS, 53 RMS), high levels of IGFBP2 and low levels of anti-IGFBP2 antibodies (cluster III: 4 HS, 50 RMS), and finally low levels of both (cluster IV: 10 HS, 7 RMS) (Figure S2). Of note, the vast majority of healthy subjects were characterized by low levels of anti-IGFBP2 antibodies (91.7%), while most of the patients (90.3%) grouped into clusters II and III, possessing higher levels of either circulating IGFBP2 protein or autoantibodies.

### 3.3. IGFBP2 and Anti-IGFBP2 Antibodies Prognostic Significance

**Figure 3:**
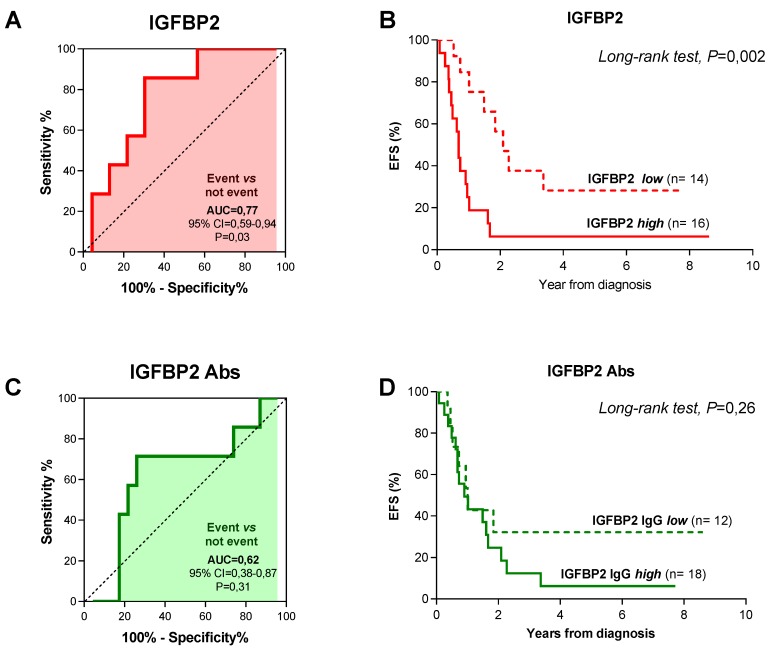
Event-free survival (EFS) analysis distinguishing metastatic RMS patients according to plasmatic IGFBP2 and anti-IGFBP2 autoantibodies levels. (**A**) ROC curve establishing the predictive value that discriminate patients experiencing an event or not based on IGFBP2 protein levels and (**B**) Kaplan-Meier survival analysis representing EFS of metastatic RMS patients distinguished according to IGFBP2 levels. (**C**) ROC curves using anti-IGFBP2 autoantibodies levels to discriminate patients as in (*A)* and (**D**) Kaplan-Meier curves showing EFS of metastatic RMS patients distinguished according anti-IGFBP2 levels. AUC, area under the curve; CI, confidence interval.

**Figure 4:**
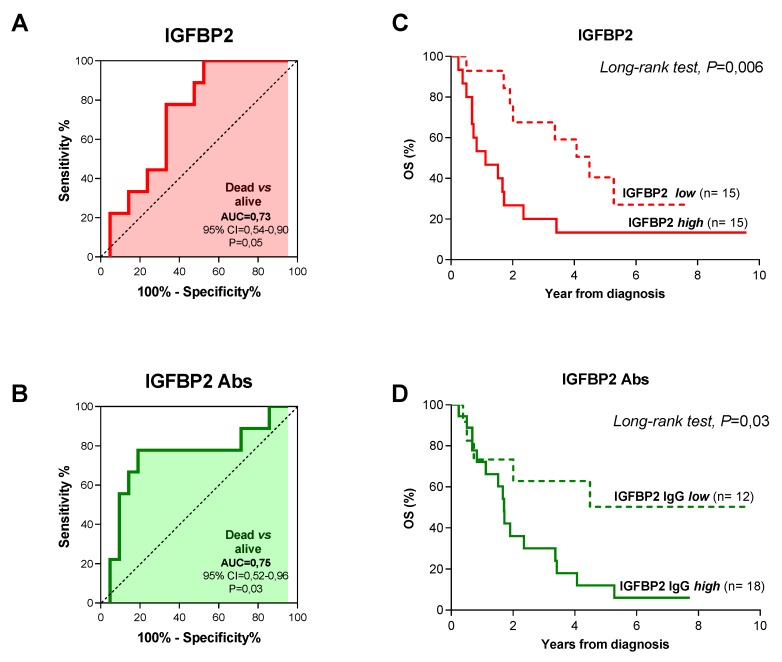
Overall survival (OS) analysis distinguishing metastatic RMS patients according to plasmatic IGFBP2 and anti-IGFBP2 autoantibodies levels. (**A**) IGFBP2 and (**B**) anti-IGFBP2 autoantibodies predictive values in metastatic RMS patients who died of disease or not. Kaplan-Meier survival analysis representing overall survival (OS) of metastatic RMS patients distinguished according to (**C**) IGFBP2 and (**D**) anti-IGFBP2 levels.

To assess whether levels of IGFBP2 and anti-IGFBP2 antibodies can predict patients’ outcome we carried out ROC curve analysis in patients for which clinical follow-up data were available (*n* = 107 patients), taking into account patient’s risk of event and overall survival. We found that neither IGFBP2 nor anti-IGFBP2 antibodies were predictive of risk of event and death in the whole patients cohort (Figure S3), likewise they failed to reach statistical significance in patients with localized disease at diagnosis (Figure S4, IRS I-III). Unlike, in metastatic patients circulating IGFBP2 protein levels were capable of discriminating patients according to event occurrence (AUC = 0.77, *p* = 0.03) and 290.55 ng/mL of IGFBP2 (sens = 85.71%; spec = 69.59%) was identified as the best cutoff value that maximized sensitivity and specificity (Figure 3A). Kaplan-Meier analysis for event-free survival (EFS), using the aforementioned cutoff value proved that RMS patients with higher IGFBP2 levels had a poorer prognosis compared to those with lower circulating protein levels (Figure 3B; long-rank test: *p* = 0.002). ROC curve obtained using anti-IGFBP2 antibody levels did not have predictive value (Figure 3C; AUC = 0.62; *p* = 0.31) and the best cutoff selected (1808 ng/mL of anti-IGFBP2; sens = 71.43%; spec = 73.91%) could not distinguish patients with different EFS (Figure 3D, *p* = 0.26). Nevertheless, combining IGFBP2 and anti-IGFBP2 cutoff values identified a group of patients with a greater risk of event. Indeed, patients with levels above the cutoffs for both IGFBP2 protein and autoantibodies had a 100% probability of experiencing an event compared to patients with levels below either one of them (63%) (Fisher’s exact test, *p* = 0.005) (Figure S5). When overall survival (OS) was considered and patients were grouped based on the optimal IGFBP2 (316,24 ng/mL; sens = 77.78%, spec = 66.67) and anti-IGFBP2 antibodies (1808 ng/mL; sens = 77.78%, spec = 80.95%) cutoff values, derived from ROC curves analysis (Figure 4A,B), we found that metastatic patients with higher values of both had less favorable prognosis compared to those with lower levels (Figure 4C,D; long-rank test: *p* = 0.005 and *p* = 0.03). Notably, all patients with both circulating IGFBP2 protein and autoantibodies levels above the cutoff values succumbed, whereas for all others OS was 55% (Figure S6; Fisher’s exact test, *p* = 0.002).

Finally, to substantiate these findings, univariate and multivariate Cox regression analysis was performed, including other clinical variables such as age, gender, histology, fusion status, tumor size, and site of onset. On univariate analysis, circulating IGFBP2 protein was the only significant variable found to be associated with reduced EFS in metastatic patients at diagnosis (*p* = 0.004; HR = 3.72; CI = 1.52−9.09) (Table 2), while both protein and autoantibodies correlated significantly with worse OS (*p* = 0.026 and *p* = 0.04) and proved to be independent negative prognostic factors in multivariate analysis (IGFBP2 *p* = 0.024; HR = 3.102; CI =1.07−9,0/anti-IGFBP2 *p* = 0.037; HR = 2.87; CI = 1.15−7.20) (Table 3).

## 4. Discussion

In childhood RMS, treatment and prognosis vary widely according to a series of clinical characteristics such as histology and fusion status, having fusion-positive RMS patients a worse outcome compared to fusion-negative ones [24,25,26]. Distal metastases remains the most adverse prognostic factor despite continuous improvement of the diagnostic capabilities and the use of more intense treatments [27,28,29]. Within this group of patients, outcome further differs significantly when multiple risk factors are combined, such as patient’s age, primary tumor site, number of metastases, and bone or bone marrow involvement [30,31]. However, individual outcome is still difficult to be predicted at the time of diagnosis. Additional risk factors, associated with treatment failure or event occurrence, are needed, to carefully stratify patients into risk groups on which to base treatment decisions and alternative front-line therapies. Blood is still an uncharted area in RMS studies despite it represents one of the most accessible source of biological material for biomarkers discovery. Blood contains circulating tumor cells, cell-free tumor DNA, and protein-based tumor antigens. Many studies have demonstrated that cancer cells can generate new antigens or deregulate expression, localization, and secretion of endogenous ones [32,33,34]. Tumor antigens are recognized by the immune system leading to the production of tumor-associated autoantibodies (TAABs). TAABs, in turn, can be used to define a unique cancer signature, as they sense changes in cellular protein expression and monitor the evolutionary dynamics of the tumor. TAABs are of particular interest as cancer biomarkers, as they can be easily isolated from blood, are more stable than antigens and their response is enduring and detectable even when triggered by a relatively small amount of antigen [35,36,37].

We have previously demonstrated that secreted insulin-like growth factor binding protein 2 (IGFBP2) is overexpressed in RMS patients and correlates significantly with tumor stage and aggressiveness [22]. Herein, we have explored the value of IGFBP2 as tumor antigen, evaluating whether IGFBP2 autoantibodies can be used either as diagnostic or prognostic biomarkers for RMS patients. In other tumor types, IGFBP2 has been reported to predict risk of relapse and resistance to therapy, with IGFBP2 autoantibodies detectable both in early stage and metastatic patients [20,38]. Namely, IGFBP2 promotes cancer cell evasion, metastasis, cancer stem cell expansion, and angiogenesis [39,40,41,42]. Its increased expression is associated with chemotherapy resistance and poor outcome. The role of IGFBP2 in RMS pathogenesis is less clear, since it affects both proliferation and survival of RMS cells by modulating NRAS expression and activity, likewise it negatively regulates IGF1R phosphorylation and signaling [16,43,44]. Our study provides the first evidence that both secreted IGFBP2 protein and autoantibodies can be detected in the blood of RMS patients at significantly higher levels than in healthy subjects and may be considered novel potential markers for RMS diagnosis. Diagnostic sensitivity of circulating anti-IGFBP2 antibodies, using the cutoff value of 2577 ng/mL, was demonstrated to be about 93%, whereas plasma levels of secreted IGFBP2 protein of 111.3 ng/mL gave a sensitivity and specificity of 90% and 73%, respectively. When IGFBP2 and anti-IGFBP2 antibody levels were combined, diagnostic sensitivity remained that high (93%), whereas anti-IGFBP2 antibody specificity improved from 50% to about 70%. Accordingly, the combination of plasmatic IGFBP2 and anti-IGFBP2 antibodies levels has already been tested in lung, glioma, and colorectal cancer patients and found to improve not only diagnostic efficacy of the test, but also its prognostic power. Concerning the possibility to use biomarkers as determinant of patients outcome, we found that blood levels of IGFBP2 protein and autoantibodies effectively distinguished children with metastatic disease at diagnosis into a more favorable and unfavorable prognosis group, whereas they did not have the same significance in patients with localized disease at diagnosis.

In the metastatic group, plasmatic IGFBP2 levels was the only factor to be associated with event occurrence in univariate analysis, while both IGFBP2 and anti-IGFBP2 antibodies levels resulted to be independent predictors of overall survival on multivariate analysis. Finally, when patients were distinguished according to IGFBP2 and anti-IGFBP2 cutoff values, event occurrence and death for patients with levels below either one of the cut-off values were 63% and 55%, respectively, whereas combining IGFBP2 and anti-IGFBP2 antibodies levels event occurrence was the highest (100%) and outcome was always fatal. Of note, our cohort was enriched of unfavorable cases, namely high risk group patients with a lower EFS and OS compared to the standard RMS population. This, however, permitted us to focus on poor prognosis patients and obtain novel prognostic indications for those children whose survival appears to be independent of both the aggressive clinical condition attributed and treatment decision assigned. Even though this is a pilot study conducted in a limited cohort of metastatic patients, certainly it could be considered a proof-of-concept to be extended to a larger cohort of metastatic patients to enforce both predictive and prognostic power of IGFBP2 protein and autoantibodies.

Taken together our study supports the importance to explore the use of liquid biopsies in rhabdomyosarcoma pediatric patients managing. We demonstrated that plasmatic levels of IGFBP2 and anti-IGFBP2 antibodies are useful biological parameters to help in the diagnosis of RMS and to predict clinical outcome in patients characterized by distal metastasis at the time of diagnosis. Based on these evidences, circulating IGFBP2 and anti-IGFBP2 autoantibodies assessment may provide a new stratification system for metastatic patients that require enforced chemotherapeutic approaches. The value of IGFBP2 and anti-IGFBP2 autoantibodies as surrogate markers for therapeutic efficacy and disease progression was not investigated in this study, by using longitudinally collected plasma samples, but it is likely since all the patients with levels above cutoff values did not respond to therapy and succumbed for progressive disease. Moreover, because of the lack of indications about the expression and clinical significance of IGFBP2 and anti-IGFBP2 antibodies in other pediatric soft tissue sarcomas, a wider analysis is warranted to unveil a role as differential diagnostic biomarkers between different types of pediatric malignancies. The clinical implications of the current study and future ones might include a new stratification of RMS patients with metastatic disease according to the levels of secreted IGFBP2 and anti-IGFBP2 antibodies and a further characterization of liquid biopsies in pediatric cancer patients.

## Figures and Tables

**Table 1:** Main clinical pathological features of 114 rhabdomyosarcoma (RMS) patients.

		# of Cases (%)
**Gender**	Male	58 (51%)
	Female	56 (49%)
**Age**	<10 years	72 (63%)
	≥10 years	42 (37%)
**Site**	Favorable *	27 (24%)
	Unfavorable *	81 (71%)
	Unknown	6 (5%)
**Size**	≤5 cm	36 (32%)
	>5 cm	67 (59%)
	Not evaluable	3 (3%)
	Unknown	8 (6%)
**IRS Group†**	I	3 (3%)
	II	9 (8%)
	III	63 (55%)
	IV	35 (30%)
	Unknown	4 (4%)
**Histology**	ARMS	50 (44%)
	ERMS	55 (62%)
	SRMS, PRMS	9 (8%)
**Fusion Status**	PAX3/7-FOXO1 ^+^	40 (35%)
	PAX3/7-FOXO1 ^−^	74 (65%)

ARMS, alveolar rhabdomyosarcoma; ERMS, embryonal rhabdomyosarcoma; SRMS, spindle cell/sclerosing rhabdomyosarcoma; PRMS, pleomorphic rhabdomyosarcoma; *FOXO1*, forkhead box protein 1; *PAX3*, paired box 3; *PAX7*, paired box 7; *PAX3/7-FOXO1+*, *PAX3/7-FOXO1* positive; *PAX3/7-FOXO1-*, *PAX3/7-FOXO1* negative. * Favorable site: orbit, urogenital non-bladder/prostate (i.e., paratesticular and vagina/uterus) and head and neck non-parameningeal; unfavorable site: head and neck parameningeal, urogenital bladder/prostate, extremities and all “other site” (i.e., thorax, abdominal, retroperitoneal, perianal, pelvis). ^†^ IRS, International Rhabdomyosarcoma Study Group. IRS group I defines completely excised tumors, group II grossly-resected tumors with microscopic residual disease, and/or regional lymph node involvement, group III gross residual disease after incomplete resection or biopsy and group IV metastatic disease.

**Table 2:** Univariate Cox regression analysis of event-free survival in 30 metastatic RMS patients.

Clinical Features		# of Cases	Univ. P-Value	HR	CI 95%
Gender	Male	14	0.283		
	Female	16			
Age	<10 years	13	0.191		
	≥10 years	17			
Site	Favorable	4	0.614		
	Unfavorable	24			
Size	≤5 cm	10	0.112		
	>5 cm	17			
IGFBP2 Abs	>1808 ng/mL	18	0.27		
	≤1808 ng/mL	12			
IGFBP2	>290,55 ng/mL	15	**0.004**	3.718	1.52–9.09
	≤290,55 ng/mL	15			
Histology	ERMS	8	0.212		
	SRMS, PRMS	2	0.668		
	ARMS	20			
Fusion Status	PAX-FOXO1 ^+^	20	0.201		
	PAX-FOXO1 ^−^	10			

ARMS, alveolar rhabdomyosarcoma; ERMS, embryonal rhabdomyosarcoma; SRMS, spindle cell/sclerosing rhabdomyosarcoma; PRMS, pleomorphic rhabdomyosarcoma; *FOXO1*, forkhead box protein 1; *PAX*, paired box; Univ., univariate analysis; HR, hazard ratio; CI, confidence interval.

**Table 3:** Uni- and multivariate Cox regression analysis for overall survival in 30 metastatic RMS patients.

Clinical Features		# of Cases	Univ. P-Value	Multiv. P-Value	HR	CI 95%
Gender	Male	14	0.163			
	Female	16				
Age	<10 years	13	0.745			
	≥10 years	17				
Tumor Site	Favourable	4	0.53			
	Unfavourable	24				
Tumor Size	≤5 cm	10	0.895			
	>5 cm	17				
IGFBP2 Abs	>1808 ng/mL	18	0.04	0.037	3.102	1.069–8.999
	≤1808 ng/mL	12				
IGFBP2	>316,24 ng/mL	15	0.026	0.024	2.872	1.146–7.20
	≤316,24 ng/mL	15				
Histology	ERMS	8	0.427			
	SRMS, PRMS	2				
	ARMS	20	0.822			
Fusion Status	PAX-FOXO1 ^+^	20	0.506			
	PAX-FOXO1 ^−^	10				

ARMS, alveolar rhabdomyosarcoma; ERMS, embryonal rhabdomyosarcoma; SRMS, spindle cell/sclerosing rhabdomyosarcoma; PRMS, pleomorphic rhabdomyosarcoma; *FOXO1*, forkhead box protein 1; *PAX*, paired box; Univ., univariate analysis; Multiv., multivariate analysis; HR, hazard ratio; CI, confidence interval

## Supplementary Materials

Supplementary materials can be found at https://www.mdpi.com/2075-4418/10/2/115/s1.

## Abbreviations

IGFBP2	Insulin-like growth factor-binding protein 2
IGF	Insulin growth factor
RMS	Rhabdomyosarcoma
ARMS	Alveolar Rhabdomyosarcoma
ERMS	Embryonal Rhabdomyosarcoma
SRMS	Spindle cell/Sclerosing Rhabdomyosarcoma
PRMS	Pleomorphic Rhabdomyosarcoma
HS	Healthy subjects
ELISA	Enzyme-Linked Immunosorbent Assay
TAA	Tumor-Associated Antigens
TAAB	Tumor-Associated Autoantibodies
CI	Confidence Interval
Yrs	Years
Abs	Antibodies
ROC	Receiver-Operator Characteristic curve
AUC	Area Under the Curve
IRS	International Rhabdomyosarcoma Study Group
EFS	Event-Free Survival
OS	Overall Survival
