# Supplementary material for: Prognostic Value of Circulating IGFBP2 and Related Autoantibodies in Children with Metastatic Rhabdomyosarcomas
Source: Diagnostics (Basel). 2020 Feb 20;10(2):115. doi: 10.3390/diagnostics10020115 (PMC7168276; doi:10.3390/diagnostics10020115)
Supplement: Supplementary file 1 [file diagnostics-10-00115-s001.pdf]

## SUPPLEMENTARY FIGURES

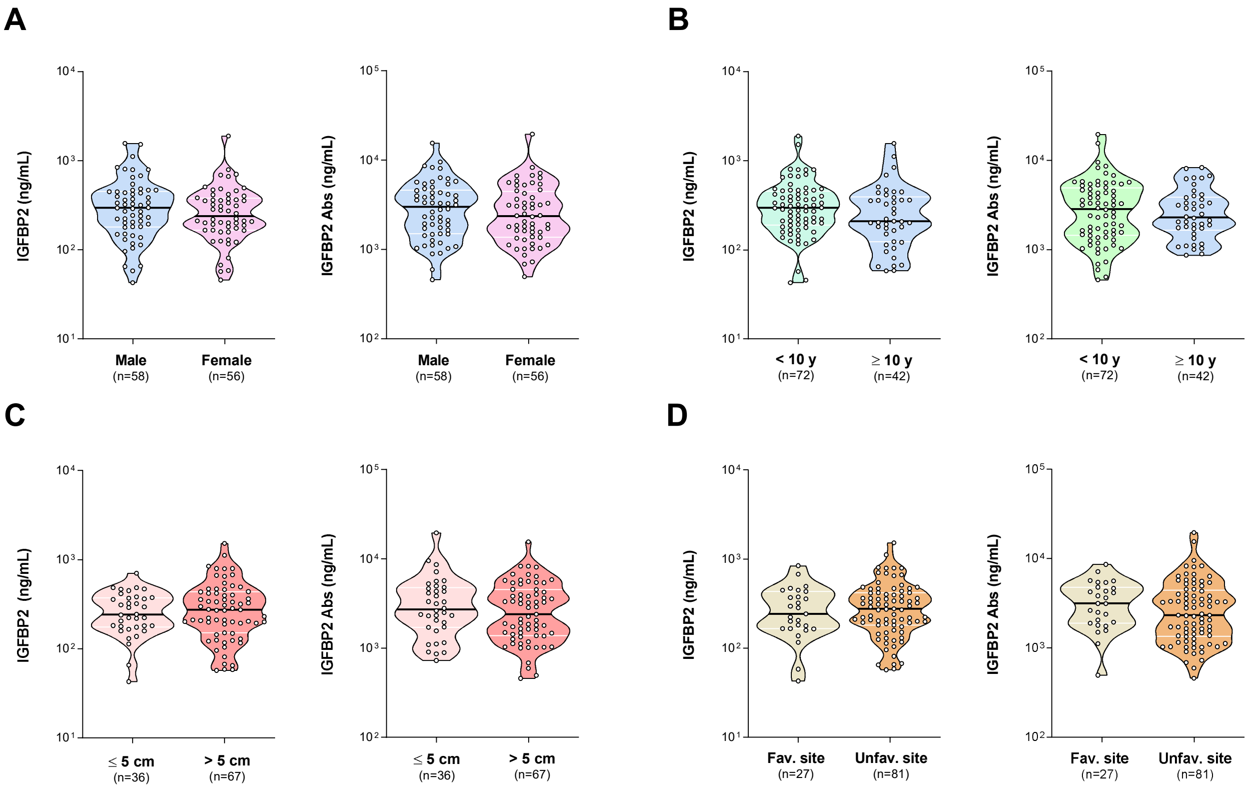


**Figure S1.** Correlation between IGFBP2 protein or autoantibodies levels and clinicophatological parameters in RMS tumors. Plasmatic IGFBP2 and anti-IGFBP2 levels assessed by direct and indirect ELISA assay were compared among groups classified by **(A)** gender, **(B)** age, **(C)** tumor size and **(D)** site of onset.

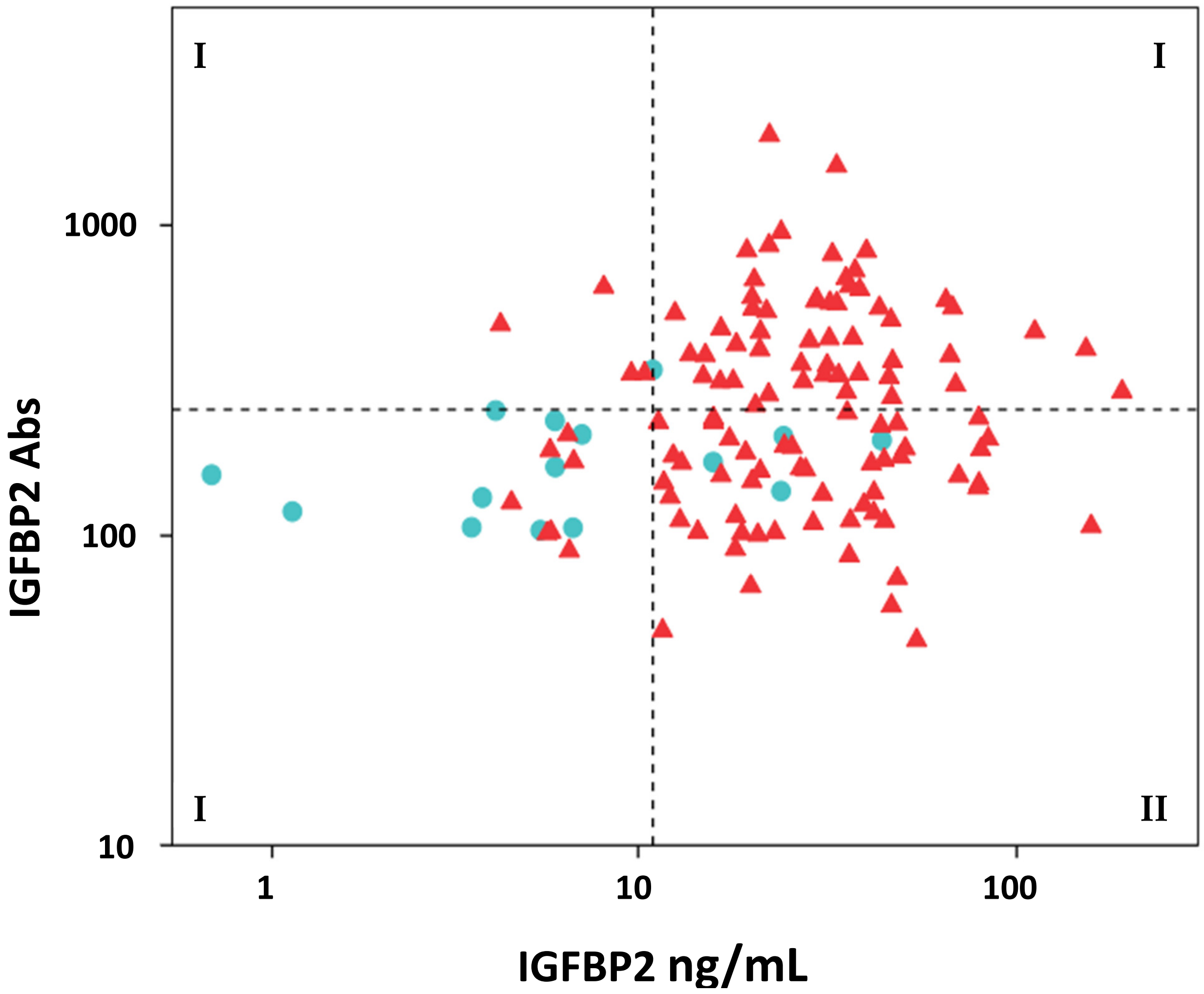


**Figure S2.** Scattergram of combined plasmatic IGFBP2 protein and anti-IGFBP2 antibodies levels in healthy subjects (●) and RMS patients (Δ). Dotted lines correspond to cut off values selected by ROC curves analysis.

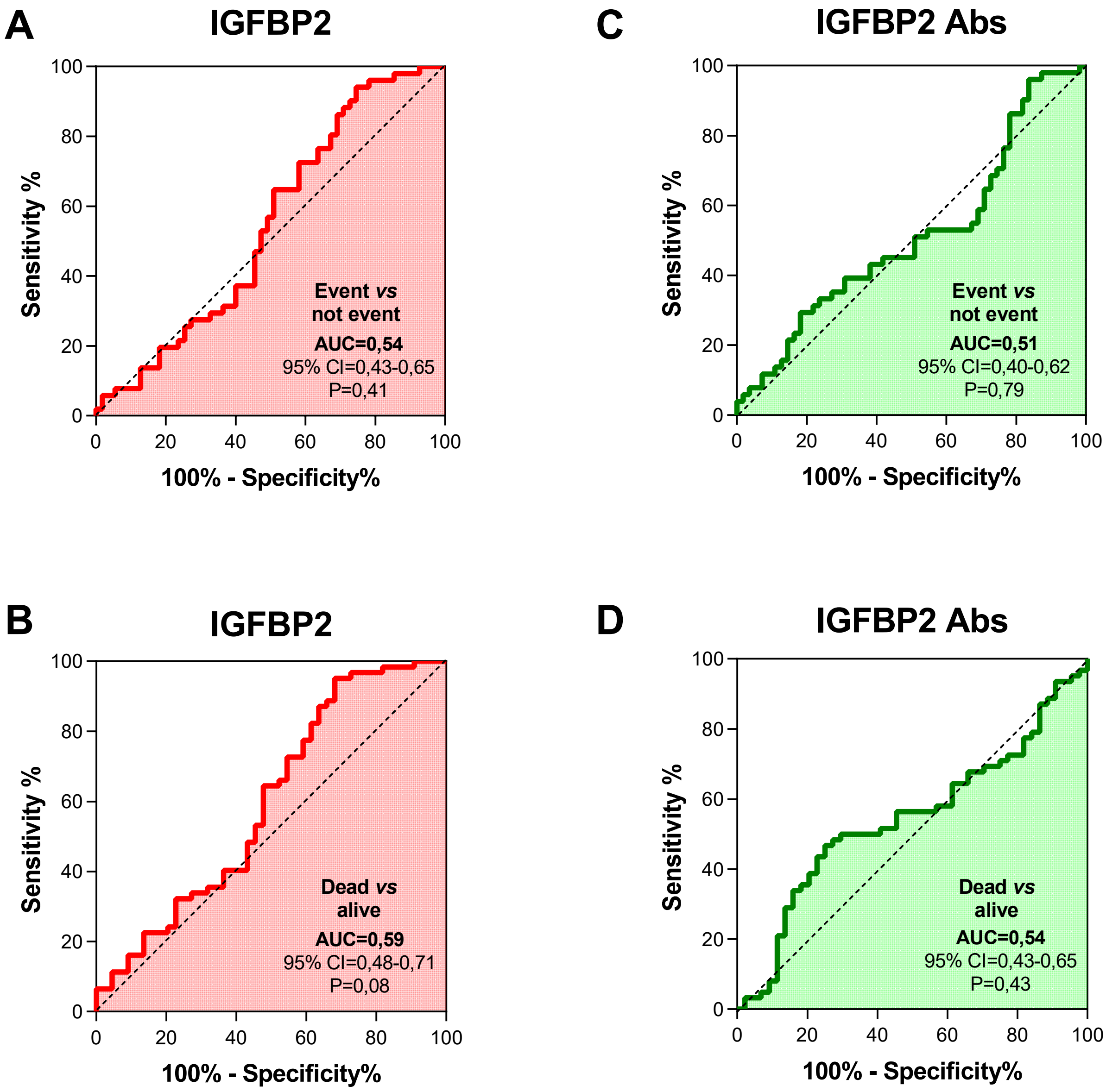


**Figure S3**. Predictive ROC analysis in RMS patients. ROCs showing the sensitivity and specificity of plasmatic IGFBP2 protein and anti-IGFBP2 antibodies as parameters to classify RMS patients according to (**A-C**) the risk of failure or (**B-D**) final outcome.

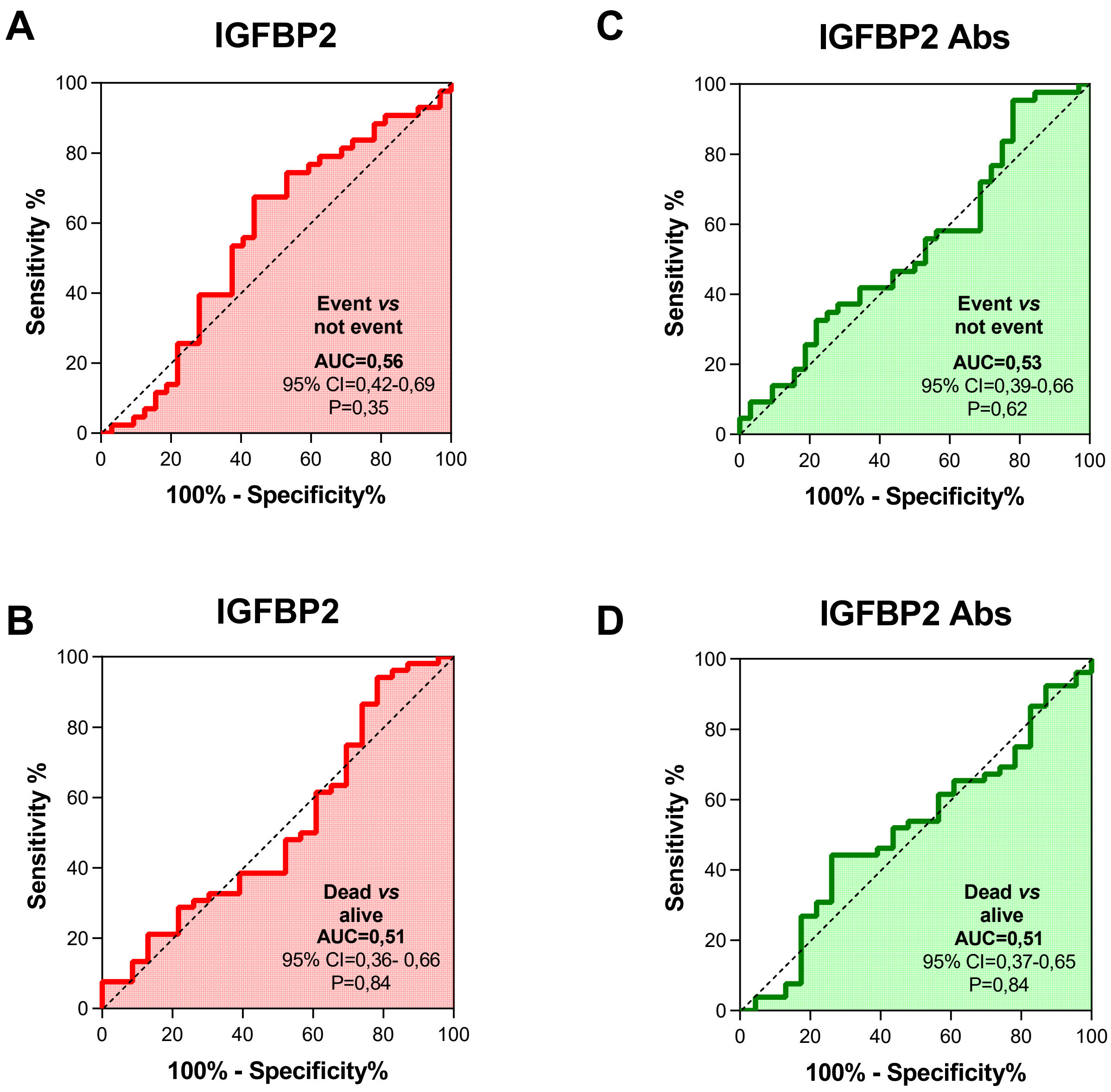


**Figure S4**. Predictive ROC analysis in RMS patients with localized disease at diagnosis. ROCs showing the sensitivity and specificity of plasmatic IGFBP2 protein and anti-IGFBP2 antibodies as parameters to classify patients according to (**A-C**) the risk of failure or (**B-D**) final outcome.

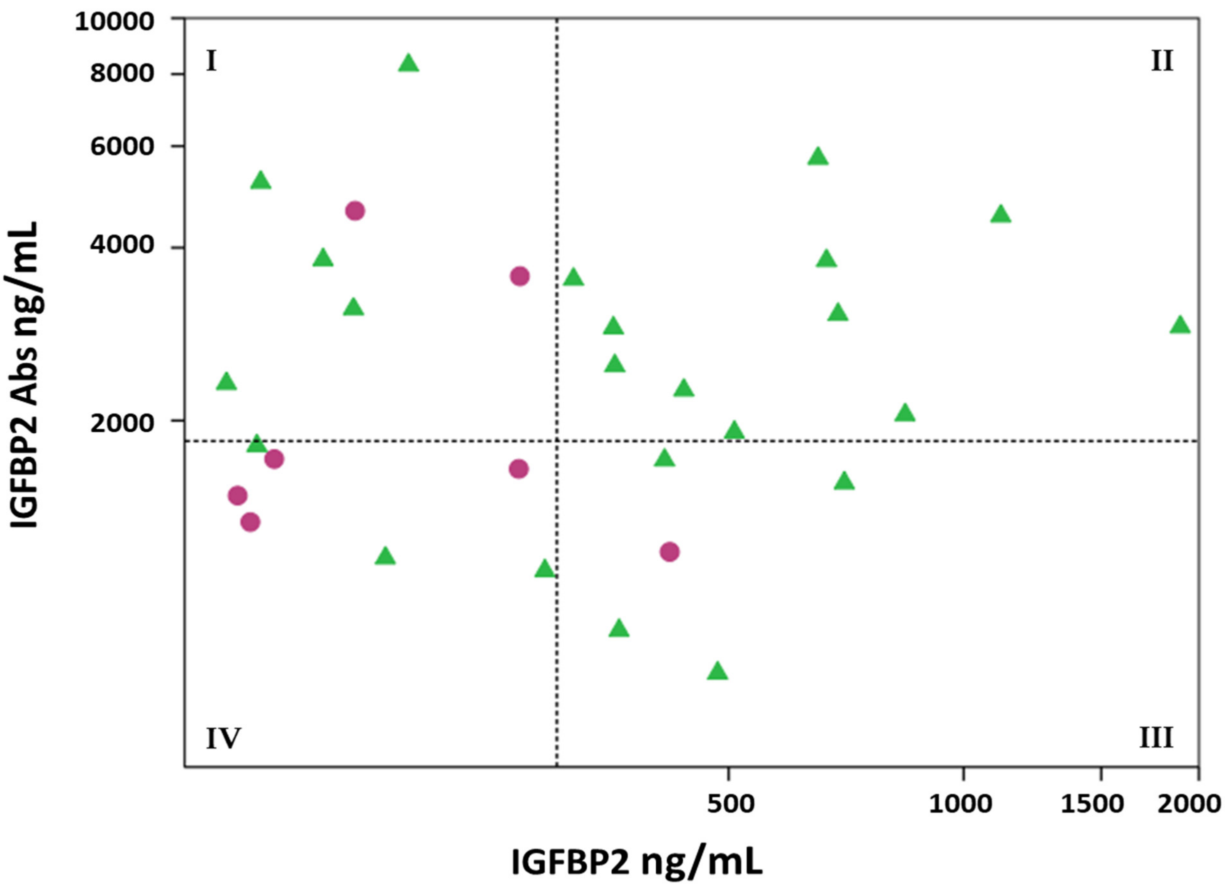


**Figure S5.** Scattergram of combined plasmatic IGFBP2 protein and anti-IGFBP2 antibodies levels in metastatic RMS patients according to risk of failure (Event *vs* not-event). Δ = event; ● = not-event. Dotted lines correspond to cut off values selected by ROC curves analysis.

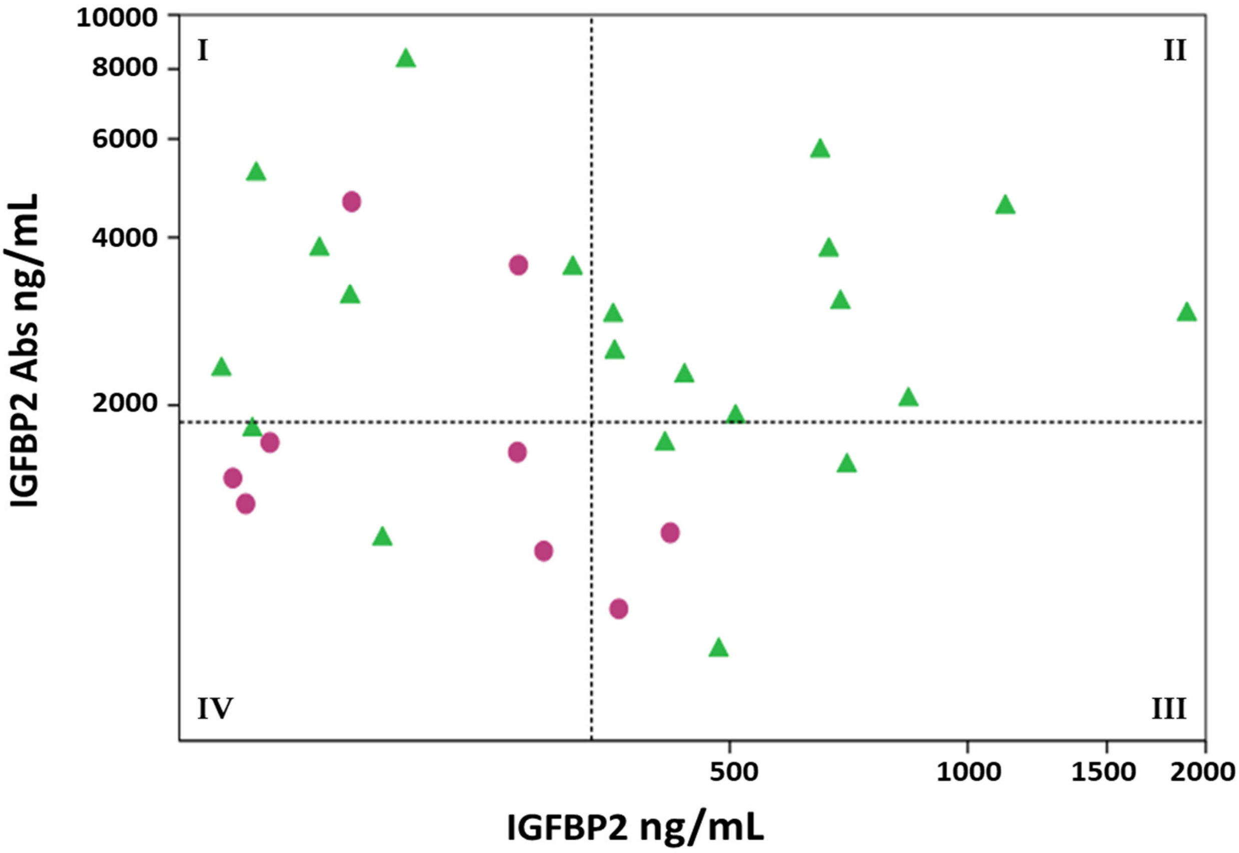


**Figure S6.** Scattergram of combined plasmatic IGFBP2 protein and anti-IGFBP2 antibodies levels in metastatic RMS patients according to final outcome (dead *vs* alive). Δ = dead; ● = alive. Dotted lines correspond to cut off values selected by ROC curves analysis.
